# Biological heterogeneity in Parkinson’s disease: implications for MRI-based patient stratification

**DOI:** 10.1038/s41531-026-01544-z

**Published:** 2026-08-27

**Authors:** Marta M. Pokotylo, Norbert Brüggemann, Jannik Prasuhn

**Affiliations:** 1 Section of Movement Disorders, Department of Neurology, University of Medical Center Schleswig-Holstein, Campus Luebeck, Luebeck, Germany; 2 Center for Brain, Behaviour, and Metabolism, University of Luebeck, Luebeck, Germany; 3 Institute of Neurogenetics, University of Luebeck, Luebeck, Germany; 4 Department of Neurology, Johns Hopkins University School of Medicine, Baltimore, MD USA; 5 F.M. Kirby Research Center for Functional Brain Imaging, Kennedy Krieger Institute, Baltimore, MD USA

**Keywords:** Biomarkers, Computational biology and bioinformatics, Neurology, Neuroscience

## Abstract

Parkinson’s disease is increasingly recognized as a heterogeneous syndrome characterized by diverse pathological processes and clinical manifestations. Here, we propose a multidimensional framework to assess this heterogeneity, with each dimension potentially captured using advanced magnetic resonance imaging techniques. The framework comprises dominant pathological processes, spatial distribution patterns of pathology across brain regions, propagation architecture, temporal dynamics of disease progression, co-pathology burden, and system response and resilience mechanisms.

## Introduction: heterogeneity as biological architecture

Parkinson’s disease (PD) has long been conceptualized as a unified clinical entity defined by cardinal motor features such as bradykinesia, rigidity, tremor, and postural instability. However, accumulating evidence reveals that PD encompasses a spectrum of distinct biological subtypes with divergent pathophysiological mechanisms, progression trajectories, and therapeutic responses^1,2^. This heterogeneity reflects fundamental differences in the disease’s underlying molecular pathways, cellular vulnerabilities, network-level dysfunction, and compensatory mechanisms^3–6^. The traditional “one-size-fits-all” therapeutic approaches fail to account for the biological and clinical presentations, potentially explaining variable treatment responses and the challenges in developing disease-modifying therapies^7–10^. Therefore, the shift from viewing PD as a homogeneous disorder to conceptualizing it as a multidimensional syndrome in which individual patients occupy distinct positions within a complex system is needed^11–14^. Emerging research presents staging systems, such as the Neuronal Alpha-Synuclein Disease Integrated Staging System (NSD-ISS) and the Synucleinopathy, Neurodegeneration, and Genetics Research Diagnostic Criteria (SynNeurGe)^15,16^. However, these systems are designed for disease diagnosis and staging and do not fully capture the heterogeneity of individual patients’ presentation.

Magnetic resonance imaging (MRI) has emerged as a non-invasive, scalable tool for characterizing distinct aspects of biological heterogeneity in PD^17–19^. MRI techniques, including neuromelanin-sensitive imaging, quantitative susceptibility mapping (QSM), diffusion tensor imaging (DTI), and volumetric analyses, provide biologically interpretable readouts that reflect distinct pathophysiological processes^20–29^. Recent data-driven approaches have leveraged multimodal MRI to identify patient subtypes characterized by distinct biological profiles and divergent clinical trajectories, with some showing rapid cognitive decline and others only motor progression^30–32^. Similarly, motor tremor-dominant versus akinetic-rigid-dominant subtypes demonstrated distinct patterns of iron deposition, neuromelanin loss, and structural connectivity alterations^33–36^, suggesting that MRI-derived biomarkers can capture biologically meaningful dimensions of heterogeneity that transcend traditional clinical classifications^6,37,38^. Importantly, heterogeneity in PD is also observed in prodromal stages, characterized by highly inter-individual heterogeneous clinical manifestations, including rapid-eye movement (REM) sleep behavior disorder (RBD), autonomic dysfunctions, depression, and hyposmia^39^. Consequently, MRI-derived biomarkers might aid in developing valuable frameworks for biologically relevant patient stratification, thereby improving clinical outcomes.

**Fig. 1:**
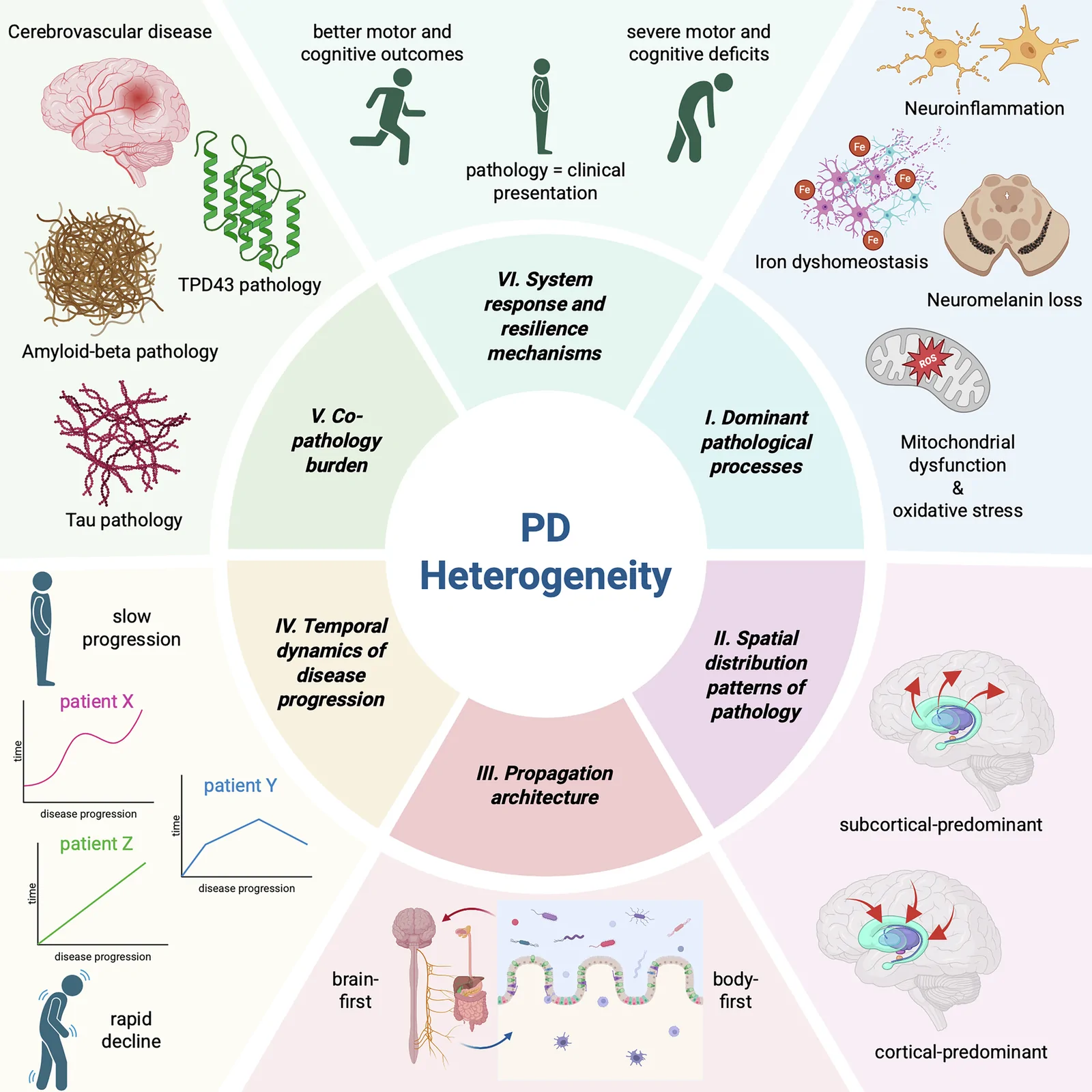
Conceptual framework of multiple dimensions underlying Parkinson’s disease heterogeneity. Six distinct dimensions underlying substantial clinical and biological heterogeneity of the disease, including (i) dominant pathological process, (ii) spatial distribution, (iii) propagation architecture, (iv) temporal dynamics of disease progression, (v) co-pathology burden, and (vi) system response and resilience mechanisms. Each dimension interacts distinctively with other dimensions in different temporal ways, with additional external factors contributing to and shaping diverse clinical trajectories. PD Parkinson’s disease; ROS reactive oxygen species; TPD-43 TAR DNA-binding protein-43.

In this perspective, we propose a multidimensional framework to conceptualize biological heterogeneity in PD that comprises six key dimensions: (I) dominant pathological processes, (II) spatial distribution patterns of pathology, (III) propagation architecture, (IV) temporal dynamics of disease progression, (V) co-pathology burden, and (VI) system response and resilience mechanisms (Fig. 1). These dimensions represent complementary biological axes along which patients vary, and each can be interrogated using specific MRI readouts and modeling approaches (Table 1)^40–43^. By integrating these dimensions, we propose a mechanistically grounded framework for characterizing PD heterogeneity that may contribute to future biomarker development, patient stratification, prognostic assessment, improved clinical trial design, hypothesis generation, and inform precision medicine strategies^44–47^. Importantly, while MRI provides scalable, biologically grounded measures of tissue integrity, spatial, temporal, and network-level alterations, it falls short of capturing molecular specificity. Therefore, the proposed framework is designed to accommodate the integration of molecular, genetic, positron emission tomography (PET) imaging, fluid, and clinical measures, enabling multimodal, multidimensional assessment and improving biological specificity and characterization accuracy.

**Table 1:** MRI modalities mapped across the six dimensions of Parkinson’s disease heterogeneity

Dimension	MRI techniques and brief description
Pathological processes	Neuromelanin-sensitive MRI - measures neuromelanin-associated contrast/volume in SNc and locus coeruleus reflecting pigment-containing neuron integrity. T2*/R2*-radiomics - captures iron-sensitive signal heterogeneity in deep nuclei^1^.
Spatial distribution patterns of pathology	T1-weighted volumetric MRI/DBM - regional cortical and subcortical volumes and deformation maps that localize atrophy patterns (neocortical, limbic, brainstem)^2^. Region-wise QSM - maps magnetic susceptibility across nuclei to localize iron deposition (SN, putamen, dentate).
Propagation architecture	Connectivity-informed epicenter models - combine structural connectivity with regional atrophy to infer putative epicenters and spread trajectories^3^. SuStaIn clustering on MRI features - derives spatiotemporal subtype stages from cross-sectional MRI metrics.
Temporal dynamics of disease progression	Longitudinal neuromelanin MRI and QSM - track progressive neuromelanin loss and iron accumulation over time in SN and connected territories^4^. Serial volumetry/DBM - quantify rates of regional atrophy across follow‑up visits^2^.
Co-pathology burden	T1 volumetry - MRI atrophy patterns to evaluate patterns suggestive of Alzheimer’s co-pathology associations^2^. Radiomics combining NM-MRI and T2* - used to detect prodromal changes and genotype differences^1^.
System response and resilience mechanisms	DTI/tractometry - fractional anisotropy (FA), axial and radial diffusivity that index white matter integrity and reserve-related microstructural changes^5,6^. Radiomics‑based network measures - individualized connectome/radiomics networks used to stratify prodromal risk and resilience patterns^7^.

This table summarizes the primary MRI techniques used to characterize each dimension of biological heterogeneity in Parkinson’s disease.

*DBM* deformation-based morphometry, *DTI* diffusion tensor imaging, *FA* fractional anisotropy, *MRI* magnetic resonance imaging, *NM-MRI* neuromelanin-sensitive magnetic resonance imaging, *PD* Parkinson’s disease, *QSM* quantitative susceptibility mapping, *SN/SNc* substantia nigra/substantia nigra pars compacta, *SuStaIn* subtype and stage interference, *T1* longitudinal relaxation time, T2*/R2* effective transverse relaxation time/effective transverse relaxation rate.

## Six dimensions of biological heterogeneity

### Dimension I – dominant pathological processes

Dominant pathological processes encompass the primary molecular and cellular mechanisms driving neurodegeneration, capturing the fundamental variability among distinct pathological cascades. MRI-based markers are currently most informative for iron dyshomeostasis and neuromelanin-associated loss, while oxidative stress, neuroinflammation, and mitochondrial dysfunction are indirectly represented through their downstream effects on tissue integrity and macrostructure (Table 2)^48–51^. Rather than simply capturing the presence of specific pathological processes, this dimension captures their relative contributions, with differences along this axis potentially underlie variability in neuronal vulnerability, clinical phenotype, progression, and therapeutic responsiveness^52–55^. The relative dominance of these mechanisms may reflect genetic variants affecting iron metabolism, mitochondrial function, or inflammatory responses, as well as environmental exposures and systemic health factors^20,27^. Understanding of dominant pathological processes provides insights into upstream biological drivers that determine downstream patterns of neuronal vulnerability, progression rates, and therapeutic responsiveness^56–59^, serving as a foundational axis that interacts with and constrains variability across other dimensions^60–63^. Importantly, disentangling the contribution of iron accumulation to PD heterogeneity is complicated by overlapping iron deposition in atypical Parkinsonian disorders and in neurodegeneration with iron accumulation, which may reflect distinct biological mechanisms. Additionally, MRI markers of iron and neuromelanin are indirect proxies for underlying processes, while the relationship between MRI markers and molecular biomarkers, such as alpha-synuclein positivity, remains poorly understood. MRI provides only macrostructural insights and lacks microscopic information on alpha-synuclein burden, neuronal loss, and synaptic dysfunction^20,64^. Therefore, MRI-based characterization provides a foundation for the multimodal integration of fluid measures, molecular biomarkers, and PET imaging to identify biologically meaningful markers of oxidative stress, mitochondrial dysfunction, neuroinflammation, and alpha-synuclein.

**Table 2:** Biological mechanisms and corresponding MRI readouts

Biological mechanism	MRI technique	Specific MRI biomarker or readout
Iron accumulation	*Direct MRI readouts*: QSM and susceptibility source separation (SSS)	Elevated magnetic susceptibility in SN, putamen, dentate nucleus; SSS isolates paramagnetic iron signal and documents progressive nigral iron spread^8^.
Neuromelanin depletion	*Direct MRI readouts*: Neuromelanin‑sensitive MRI	Reduced NM contrast‑to‑noise ratio and normalized NM volume in SNc and LC; regional NM loss correlates with motor and cognitive severity^1^.
Neuroinflammation	*Indirect MRI proxies*	Diffusion MRI – increased free-water reflective of extracellular water accumulation associated with neuroinflammation processes in SN^21,74^. Proton density MRI – reflective of non-specific changes in tissue water content, with not sufficient evidence in SN^75^.
Alpha‑synuclein pathology	*Indirect MRI proxies*	MRI atrophy subtypes map onto neuropathological Lewy distributions (brainstem, limbic, neocortical) but MRI does not directly detect alpha‑synuclein; MRI atrophy patterns are used as anatomic proxies for spread^2^.
Dopaminergic denervation	*Indirect MRI proxies*	Early striatal dysfunction precedes nigral iron and NM changes; NM‑MRI and QSM changes in nigrostriatal territories reflect dopaminergic innervation involvement^4^.
White matter degeneration	*Direct MRI readouts*: DTI/tractometry	Reduced FA, AD, and RD in PD subtypes (notably diffuse‑malignant), with tract‑specific FA/RD correlating with motor and cognitive scores^5,6^.
Cortical atrophy	*Direct MRI readouts*: T1 volumetry/deformation‑based morphometry (DBM)	Regional cortical thinning and volume loss (frontal, parietal, temporal, precuneus) with subtype‑specific atrophy trajectories and accelerated rates in diffuse‑malignant PD^2,9^.
Network dysfunction	*MRI-based modeling*: Connectivity/epicenter modeling and radiomics networks	Epicenter‑driven atrophy patterns and individualized radiomics‑based brain networks differentiate subtypes (cerebellar/midbrain vs cortical/striatal epicenters) and predict progression^3,7,10^.

This table links key pathological mechanisms in PD to specific MRI techniques and imaging biomarkers.

*AD* axial diffusivity, *DBM* deformation-based morphometry, *DTI* diffusion tensor imaging, *FA* fractional anisotropy, *LC* locus coeruleus, *MRI* magnetic resonance imaging, *NM* neuromelanin, *NM-MRI* neuromelanin-sensitive magnetic resonance imaging, *PD* Parkinson’s disease, *QSM* quantitative susceptibility mapping, *RD* radial diffusivity, *SN (SNc/SNr)* substantia nigra (pars compacta/pars reticulata), *SSS* susceptibility source separation, *T1* longitudinal relaxation time.

### Dimension II – spatial distribution patterns of pathology

The spatial distribution patterns of pathology represent the anatomical patterns and regional selectivity of pathological changes across the brain in PD (Table 3)^1^. Patients differ in the topographic signature of neurodegeneration, with some exhibiting pathology concentrated in the substantia nigra and basal ganglia (subcortical-predominant pattern), others showing extensive cortical atrophy involving the frontal, temporal, and parietal regions (cortical-predominant pattern), and still others demonstrating intermediate/mixed patterns. Spatial distribution patterns correlate with distinct clinical phenotypes, such as motor-predominant versus cognitive-predominant, reflecting underlying differences in network vulnerability and propagation pathways, and interact with the propagation architecture to determine the disease trajectory^6,29,37^. Yet we lack a mechanistic understanding of the factors that determine whether some patients develop cortical-predominant versus subcortical-predominant patterns. The lack of comprehensive longitudinal evidence on the stability or change of these spatial patterns over the clinical lifetime further limits our understanding. Additionally, the spatial distribution of pathology overlap between PD and Parkinsonian syndromes further complicating clinical characterization, prompting validation of improved imaging markers. However, these uncertainties may be partially addressed through a comprehensive assessment of MRI-based macrostructure, combined with PET imaging, fluid, and molecular biomarkers, to further validate the biological drivers of spatial differences and their roles in disease progression.

**Table 3:** Examples of spatial distribution patterns and related data-driven classification in Parkinson’s disease

Spatial subtype	Anatomical regions affected	MRI findings
Brainstem‑predominant	Brainstem (SN, LC), rostral brainstem structures, later amygdala/hippocampus	*Direct MRI readouts:* Early brainstem atrophy on T1/DBM; early NM loss in SN/LC; progressive rostral extension on serial MRI^2,4^.
Limbic‑predominant	Amygdala, accumbens, striatum, temporal cortex	*Direct MRI readouts:* Early limbic and striatal volume loss on T1; later spread to parietal and frontal cortices^2^.
Neocortical/cortex‑first	Frontal and parietal neocortex early	*Direct MRI readouts:* Early cortical thinning, volume loss on T1; cortex‑first subtype observed with higher RBD prevalence in one cohort^2,11^.
Diffuse‑malignant	Widespread cortical and subcortical involvement including precuneus, inferior temporal, fusiform, lateral cerebellum	*Direct MRI readouts:* Accelerated multi‑regional atrophy (DBM) and worse longitudinal decline in motor, cognition, and ADL; more rapid progression on serial MRI^2,9^.
Restricted or deep‑grey matter first	Predominantly deep grey nuclei (SN, striatum, pallidum) with relative cortical sparing early	*Direct MRI readouts:* Deep‑grey first subtype shows early nigrostriatal/striatal involvement on volumetry/QSM; associated with different symptom clusters and milder early cortical symptoms^11,12^.
Symmetric vs asymmetric degeneration	Lateralized onset reflects asymmetric nigral involvement vs bilateral symmetric patterns.	*Direct MRI readouts:* Hemispheric asymmetry in NM loss and iron accumulation contralateral to motor onset shown by NM‑MRI and QSM^13^.

This table describes MRI-defined spatial subtypes of PD based on data-driven clustering and staging algorithms.

*ADL* activities of daily living, *DBM* deformation-based morphometry, *LC* locus coeruleus, *MRI* magnetic resonance imaging, *NM* neuromelanin, *PD* Parkinson’s disease, *RBD* rapid-eye movement sleep behavior disorder, *SN* substantia nigra, *T1* longitudinal relaxation time.

### Dimension III – propagation architecture

The propagation architecture dimension represents the spatiotemporal trajectory, directionality, and network organization through which pathological processes may spread, capturing differences in where the disease begins and how it progresses through the nervous system (Table 4). Along this dimension, patients may vary in the anatomical origin of pathology, the sequence of regional involvement, and the connectivity pathways through which pathology propagates^24,26^. This dimension may distinguish between brain-first versus body-first trajectories, reflecting the central or peripheral site of initiation of alpha-synuclein pathology. However, ongoing research aims to determine whether these categories represent distinct PD ‘types’ or rather the extreme ends of a continuous spectrum, as propagation architecture has vast clinical implications by determining the temporal ordering of motor and non-motor symptoms, and influences disease progression rates. Divergence in propagation architecture is driven by mechanisms involving alpha-synuclein strain variability, network connectivity, and selective vulnerability. As the propagation architecture interacts with temporal dynamics to determine progression velocity, our limited understanding of how stable it remains or how it changes over time further complicates accurate patient stratification and clinical progression predictions^6,37^. Importantly, MRI provides indirect insights into propagation trajectories and cannot independently verify disease origin. Therefore, it should be interpreted alongside complementary measures of autonomic function, longitudinal clinical observation, and alpha-synuclein amplification assays.

**Table 4:** Proposed propagation architecture models in Parkinson’s disease and associated MRI correlates

Model	Key MRI features	MRI interpretation	Clinical characteristics
Brain‑first	*Exploratory markers:* Early cortical or brainstem atrophy on T1; early NM loss in SN/LC; subtype patterns identification using clustering approaches and SuStaIn^2,11^.	May be consistent with proposed central initiation, but cannot identify original place of pathology initiation.	Presents with central non‑motor symptoms or early cognitive involvement in cortex‑first; progression varies with subtype but may show earlier cognitive decline^2^.
Body‑first	*Direct MRI readouts:* Earlier brainstem (LC, SN) involvement and later limbic, cortical changes in staging models^12^.	May reflect correlates of proposed peripheral-to-central propagation.	Associated with RBD and autonomic dysfunction early; may have poorer levodopa response and worse prognosis.
Connectivity epicenter models	*Direct MRI readouts:* Epicenter‑based atrophy trajectories derived from MRI plus connectome predict spread from cerebellar, midbrain or cortical, striatal epicenters^3,10^.	Provides indirect evidence of connectivity-mediated spread.	Epicenter patterns link to faster motor progression (cerebellar, midbrain epicenter) versus milder progression (cortical, striatal epicenter) and overlap with other movement disorders in one subtype^3,10^.
Disease reservoir models	*Exploratory markers:* Cortical thinning propagates from “disease reservoir” and is greater in regions with stronger functional connectivity as defined by DBM^76,77^.	May suggest possible network-mediated disease propagation; requires longitudinal validation.	Connectivity propagation patterns may predict future cognitive decline^77^.
Epidemic reservoir models	*Exploratory markers:* SIR model-based prediction of spatial and temporal patterns of atrophy seeding from SN^78,79^.	MRI insights are used for a theoretical modeling of propagation patterns.	Model based estimation of spatial and temporal progression of PD.

This table outlines different models of pathological spread in PD and MRI approaches that may support these hypotheses.

*DBM* deformation-based morphometry, *LC* locus coeruleus, *MRI* magnetic resonance imaging, *NM* neuromelanin, *PD* Parkinson’s disease, *RBD* rapid eye movement sleep behavior disorder, *SIR* Susceptibility-Infected-Removed, *SN* substantia nigra, *SuStaIn* subtype and stage interference, *T1* longitudinal relaxation time.

### Dimension IV – temporal dynamics of disease progression

**Fig. 2:**
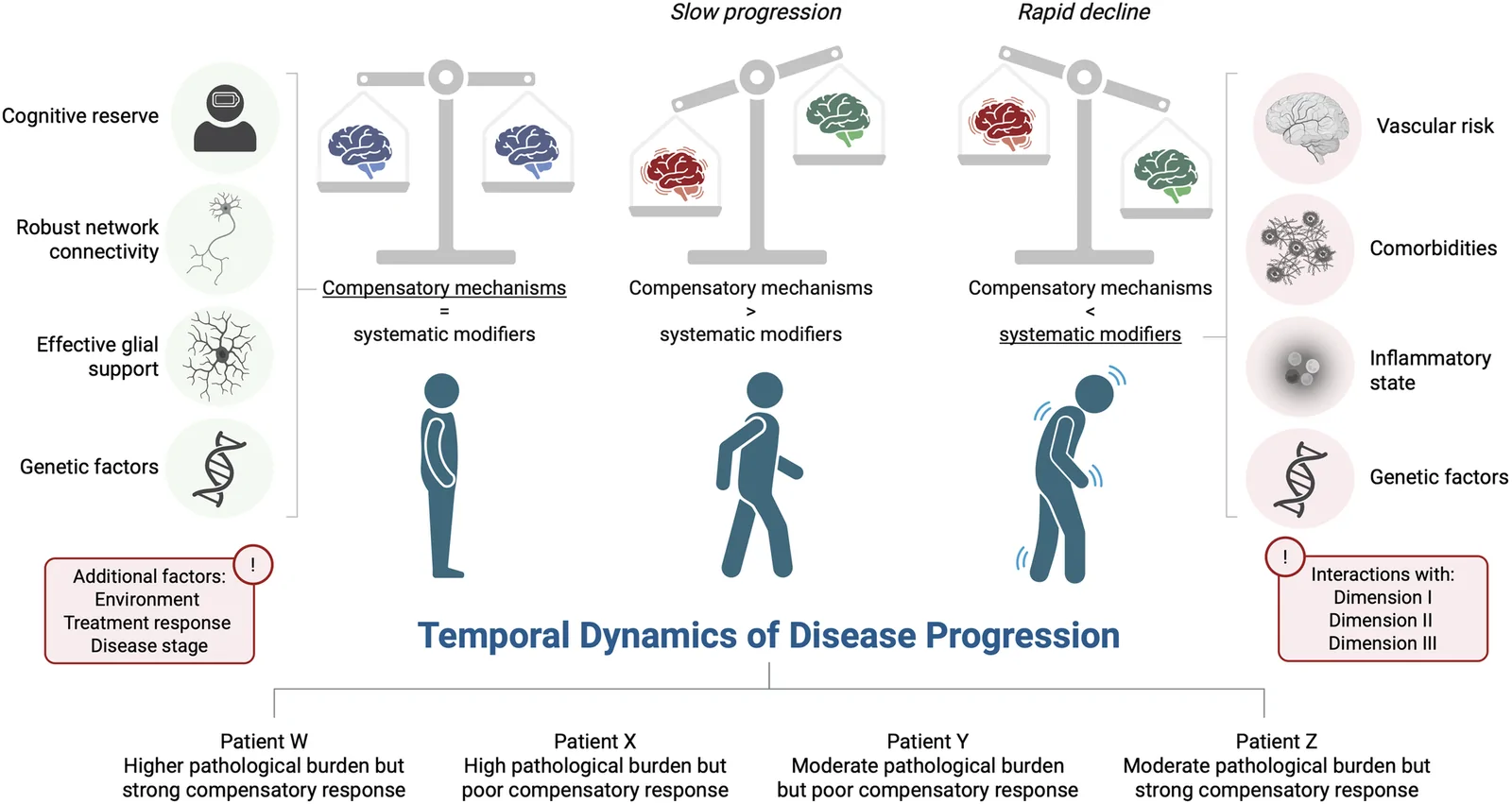
Conceptual model of dimension IV: temporal dynamics of disease progression. Schematic representation of the complex relationship between compensatory mechanisms and systematic modifiers’ influence on the temporal dynamics of Parkinson’s disease progression. Given its complex interactions, there is a high degree of inter-individual variability in disease trajectories, with additional factors such as environment, treatment responses, and disease stage contributing to intra-individual differences. Temporal dynamics also interact with other dimensions, such as (I) dominant pathological processes, (II) spatial distribution patterns of pathology, and (III) propagation architecture within each patient, making Parkinson’s disease even more heterogeneous.

The temporal dynamics of disease progression represent the rate and pattern of disease progression over time, capturing variability in how rapidly pathological changes accumulate and in how rapidly clinical symptoms worsen (Fig. 2). This dimension distinguishes between slow progressors, who maintain relatively stable function over years, and fast progressors, who experience rapid motor and cognitive decline^1^, as well as the temporal profile of whether decline is linear, accelerating, or characterized by periods of stability punctuated by rapid worsening^25,50,57,64^. Importantly, temporal dynamics focus on the evolution of pathological changes rather than their clinical expression, as clinical progression also reflects compensatory mechanisms, pathology burden, and the sensitivity of clinical assessment, resulting in a mismatch between the extent of pathological changes and clinical presentation. Divergence in the temporal dynamics is driven by multiple interacting mechanisms, including intrinsic toxicity of dominant pathological processes, compensatory mechanisms, genetic factors, and systemic modifications (vascular risk factors, inflammatory states, and comorbidities). The main difficulty lies in disentangling the relative contributions of underlying dominant pathological processes, spatial distribution, and propagation architecture of the pathology, and therapeutic interventions to the observed rate of disease progression. As clinical measures do not directly reflect biological progression, and we lack meaningful longitudinal datasets that capture imaging, molecular, and clinical changes, improved multimodal approaches are necessary. Therefore, longitudinal MRI integration with fluid biomarkers, PET imaging, and comprehensive clinical assessment may partially address the issue and improve the identification of biologically integrated and clinically meaningful temporal trajectories.

### Dimension V – co-pathology burden

The co-pathology burden dimension represents the extent and type of additional proteinopathies and pathological processes that coexist with alpha-synuclein pathology. This dimension captures variability in the presence, severity, and spatial distribution of tau pathology, amyloid-beta plaques, TAR DNA-binding protein 43 (TDP-43) inclusions, cerebrovascular disease, and other age-related pathologies (Table 5)^60–63^. Co-pathologies profoundly influence clinical phenotype, progression rates, and treatment responsiveness, often determining whether patients develop dementia and how rapidly they decline^30–32^. Co-pathologies influence spatial distribution patterns, determining regional vulnerability; temporal dynamics of disease progression influence progression rates; and system response and resilience mechanisms modulate compensatory capacity^6,37,38^. However, the extent of co-pathologies’ contributions to each aspect of heterogeneity in PD remains unresolved, as we lack meaningful tools. Additionally, genetic factors and systemic modifiers, including vascular risk factors and inflammatory states, promote cerebrovascular pathology and may accelerate multiple proteinopathies, explaining some variability among patients^65–67^. However, MRI lacks molecular specificity and provides approximations of various molecular pathologies, which require further validation. MRI-derived markers of hippocampal atrophy, vascular injury, and cortical thinning should be combined with tau and amyloid PET imaging, genetic profiling, cerebrospinal fluid biomarkers, and neuropathological evidence to better characterize co-pathologies. Additionally, ^18^F-fluorodeoxyglucose (^18^FDG)-PET may provide insights into regional energy utilization, contributing to the co-assessment of co-pathologies and their functional consequences. Importantly, such a multimodal assessment is necessary in a longitudinal setting to gain insights into the temporal interactions between dominant pathological processes and co-pathologies, and the resulting effects on clinical manifestations.

**Table 5:** MRI markers of co-pathology burden in Parkinson’s disease

Co‑pathology	MRI assessment method	Specific imaging markers	Interpretation and relevance
Alzheimer’s disease pathology (amyloid‑β)	*Indirect proxies:* MRI volumetry, DBM	Regional neocortical atrophy (frontal, parietal) associated with subgroup classification^2^.	May indicate concomitant AD-related pathology, but requires amyloid/tau PET or fluid biomarker validation^2^.
Cerebrovascular disease	*Direct MRI readouts:* Structural MRI (T2, SWI)	WMH, cerebral microbleeds and enlarged perivascular spaces^80^.	Reflects vascular injury burden associated with cognitive decline and gait dysfunction in PD^80,81^.
Parkinson’s disease with dementia, Dementia with Lewy Bodies	*Indirect proxies:* MRI volumetry & VBM	Atrophy of frontal, temporal lobes, and entorhinal cortex^82,83^.	Inconsistent and conflicting evidence of frontal and temporal lobes atrophy associating with cognitive impairment lacking specificity^82,83^.
Rapid-eye movement behavioral sleep disorder	*Indirect proxies:* MRI volumetry, structural MRI	Inferior temporal thinning, insular and caudate atrophy^84^.	May identify patients with mode diffuse neurodegeneration and higher risk of cognitive impairment.
Other neurodegenerative changes (non‑Alzheimer’s disease)	*Exploratory markers:* Multimodal MRI (T1, DTI, QSM)	Regional atrophy, white matter microstructural damage (reduced FA, increased RD), and iron abnormalities^5,9^.	Identified MRI changes are non-specific and required multimodal validation using PET, genetic, and molecular biomarkers.

This table outlines approaches for detecting and quantifying co-pathologies in PD using MRI and complementary techniques.

*AD* Alzheimer’s disease, *DBM* deformation-based morphometry, *DTI* diffusion tensor imaging, *FA* fractional anisotropy, *MRI* magnetic resonance imaging, *PD* Parkinson’s disease, *PET* positron emission tomography, *QSM* quantitative susceptibility mapping, *RD* radial diffusivity, *SWI* susceptibility-weighed imaging, *T1* longitudinal relaxation time, *T2* effective transverse relaxation time, *VBM* voxel-based morphometry, *WMH* white matter hyperintensities.

### Dimension VI – system response and resilience mechanisms

**Fig. 3:**
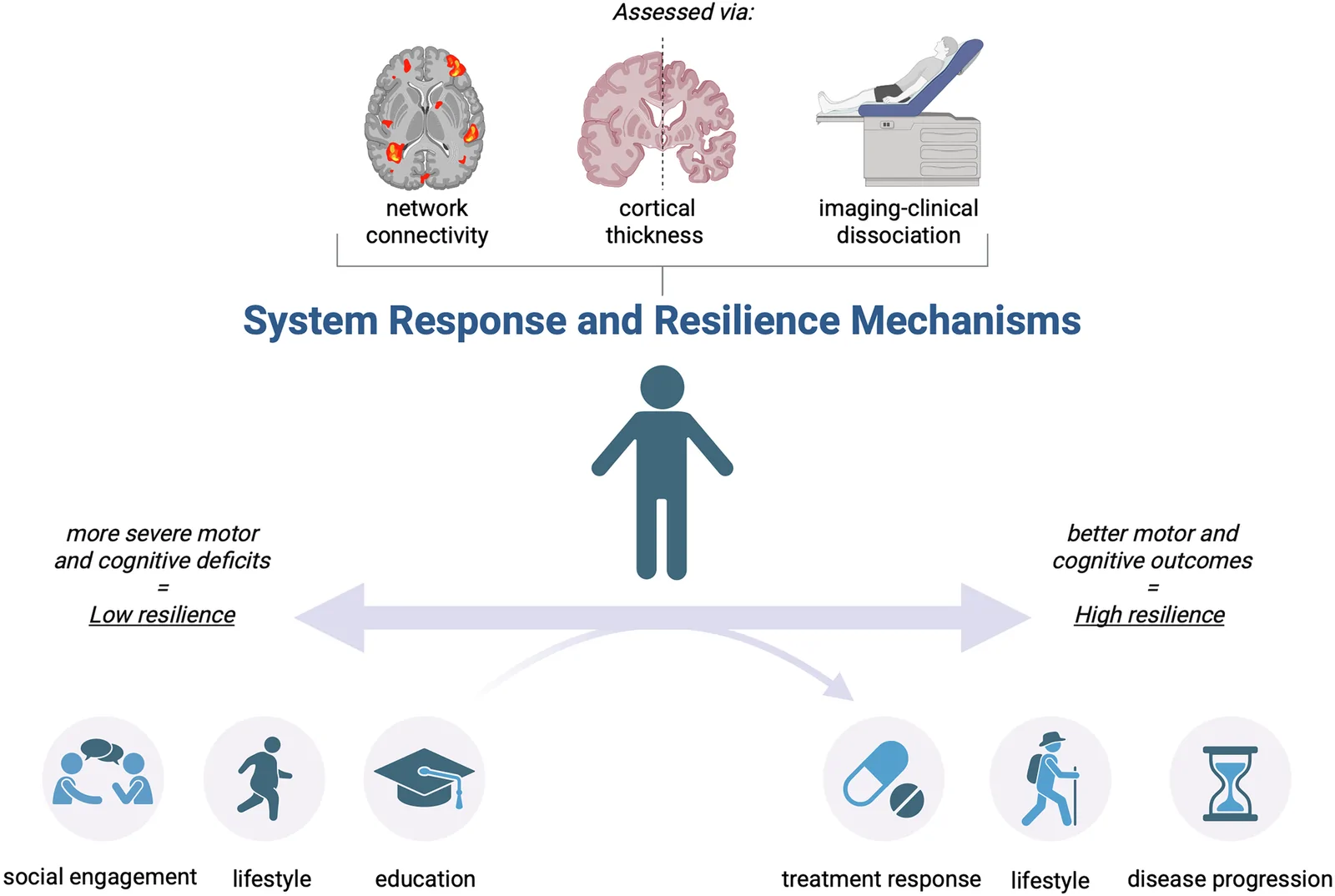
Conceptual model of dimension VI: system response and resilience mechanisms. System response can be evaluated via biologically interpretable magnetic resonance imaging features such as network connectivity, cortical thickness, and imaging-clinical dissociation. The inter-individual system responses place the patient on the resilience spectrum, with additional factors such as social engagement, education, and lifestyle contributing. In turn, system resilience contributes to the heterogeneity in patients’ treatment responses, lifestyles, and disease progression. System response and resilience interact with other dimensions, such as (I) the dominant pathological processes, (II) spatial distribution patterns of pathology, (III) propagation architecture, (IV) temporal dynamics of disease progression, and (V) co-pathology burden within each patient, making Parkinson’s disease even more heterogeneous.

The system response and resilience mechanisms dimension represents the brain’s capacity to maintain function despite accumulating pathology through compensatory mechanisms, network reorganization, and adaptive responses. It captures variability in how effectively individual patients buffer the clinical impact of neurodegeneration, ranging from prodromal patients who do not convert to the symptomatic stage, those who maintain relatively preserved function despite substantial pathology (high resilience), to those who exhibit severe symptoms despite minimal pathological burden (low resilience) (Fig. 3)^63,68^. Resilience mechanisms may determine prognosis, influence quality of life, and represent potential therapeutic targets to enhance compensatory capacity in PD^30–32^. Importantly, this dimension characterizes biological resilience and compensatory mechanisms rather than defines a resilient patient phenotype; therefore, indirect MRI markers rely on imaging-clinical dissociations rather than direct visualization of compensatory mechanisms^20,64^. However, divergence along this dimension is driven by multiple mechanisms, including cognitive reserves, brain reserves, network reorganization, synaptic plasticity, glial support mechanisms, genetic factors, and systematic modifiers, which are not directly assessable with MRI. System response and resilience mechanisms may modulate the clinical impact of dominant pathological processes, determine how spatial distribution patterns of pathology translate into functional deficits, influence the propagation architecture, shape the temporal dynamics of disease progression, and buffer the effects of co-pathologies^6,37^. Most importantly, the balance between pathological burden and compensatory capacity determines individual clinical trajectories, with high-resilience patients maintaining function longer despite progressive pathology^63,68^. Despite the promising clinical implications, our understanding of the factors determining why some patients exhibit high resilience while others show low resilience remains incomplete. Further research using multimodal longitudinal approaches is necessary to address the shortcomings of isolated MRI use and better link structural and clinical associations in PD. For instance, the relationship between MRI-detected network reorganization and actual compensatory function requires validation using task-based functional imaging and behavioral studies^29^.

**Fig. 4:**
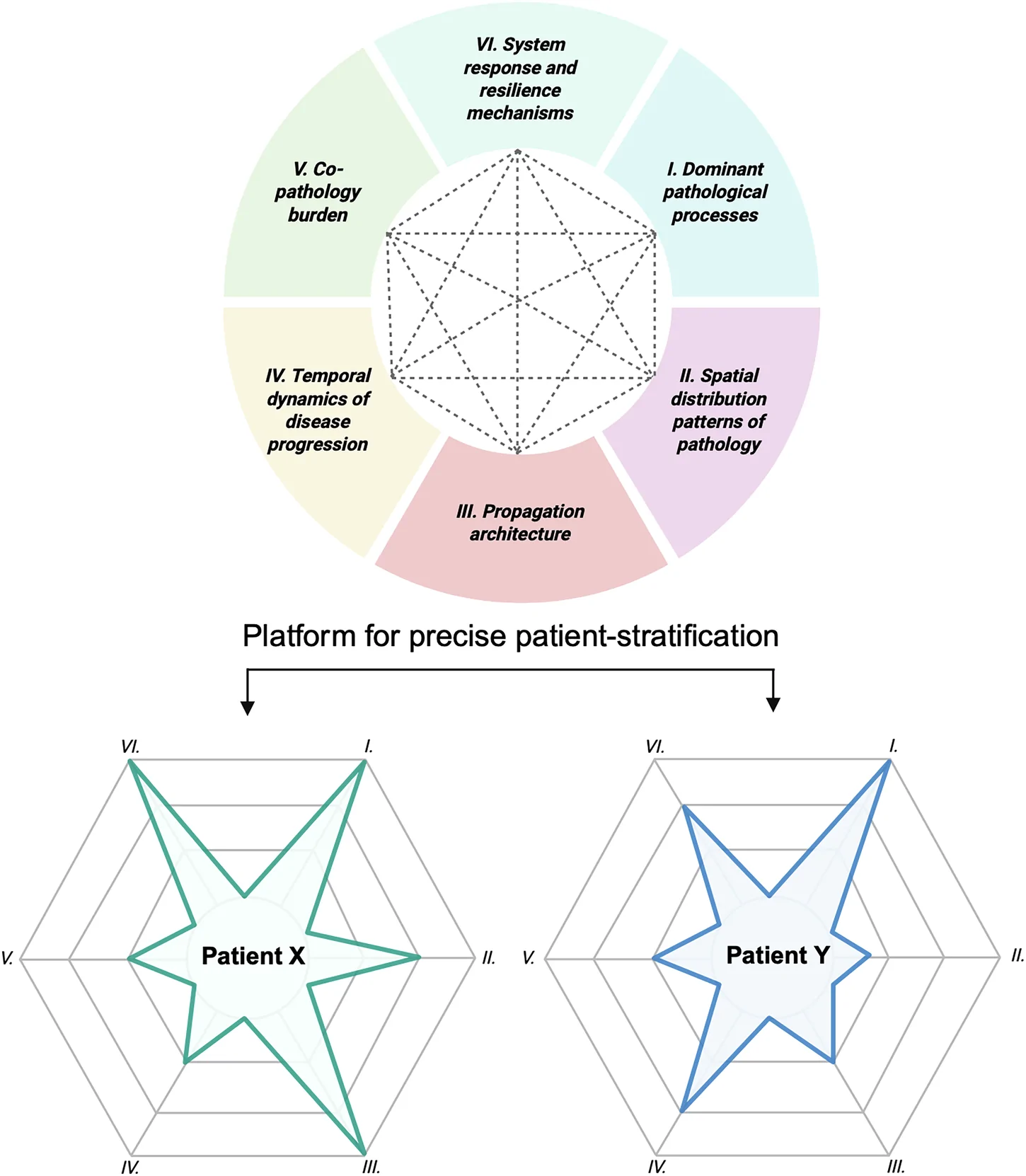
Schematic representation of a multidimensional patient stratification framework in Parkinson’s disease. The key six dimensions contributing to the diverse clinical trajectories of Parkinson’s disease are (I) the dominant pathological processes, (II) the spatial distribution patterns of pathology, (III) the propagation architecture, (IV) the temporal dynamics of disease progression, (V) the co-pathology burden, and (VI) the system response and resilience mechanisms. These dimensions can be integrated into a multidimensional biological and clinical profile, including each factor’s relative contribution (as demonstrated by radar plots of patients X and Y), providing a foundational framework for developing biologically informed patient stratification and precision medicine approaches.

The six outlined dimensions represent a continuous biological axis rather than discrete disease categories, with individual patients occupying distinct positions along each dimension, thereby generating a multidimensional biological profile (Fig. 4). Within this framework, discrete subtypes such as cortex-first, deep-gray-first, body-first, or brain-first may emerge as estimates of recurring patterns rather than as distinct entities.

## MRI as a primary tool for biological phenotyping of Parkinson’s disease

MRI is a scalable, non-invasive, and importantly, a widely accessible approach for multidimensional characterization of PD heterogeneity. Although MRI provides varying degrees of specificity across the six proposed dimensions, it serves as a common framework for assessing PD heterogeneity in vivo. Above all, MRI provides a meaningful foundation and should be integrated with other modalities to create a broader biological framework.

QSM can be used for iron quantification, neuromelanin-sensitive MRI for dopaminergic neuronal integrity, and relaxometry measures (e.g., R2^*^) capture the combined effects of iron and brain microstructure effects^54,64,69,70^. Dimensional profiling can characterize patients’ positions along the iron-neuromelanin axis, while clustering methods may identify subgroups with distinct pathological profiles, potentially identifying individuals who may benefit from mechanism-targeted therapies^9,71^.

Regional volumetric measures of gray matter atrophy, cortical thickness maps revealing laminar-specific degeneration, and subcortical shape analyses detecting focal atrophy enable analysis of the spatial distribution of pathology^20–23^. Voxel-based morphometry and surface-based analyses provide whole-brain spatial maps of structural alterations, while neuromelanin-sensitive MRI and QSM capture spatial patterns of neuromelanin and iron content in subcortical regions^24,26,28,29^. Dimensional profiling may position patients along a cortical-subcortical axis and identify individuals at risk of specific clinical trajectories. Clustering methods may aid characterization of discrete spatial subtypes, including “cortex-first” versus “deep gray matter-first” progression patterns^28^, while trajectory modeling, e.g., using subtype- and stage-inference (SuStaIn) algorithms, can reconstruct the temporal sequence of regional involvement, revealing a distinct spatial progression pathway^24,26^.

The propagation architecture can be assessed with sequential patterns of regional involvement detected through longitudinal volumetric analyses, connectivity-based measures that reveal network-level propagation pathways, and brainstem-specific markers that distinguish brain-first from body-first subtypes^20–23^. Neuromelanin-sensitive MRI of the brainstem may identify patterns consistent with brain-first subtypes, while measures of autonomic function, including cardiac imaging, may identify patterns consistent with body-first subtypes^61,68,72,73^. However, these insights should be interpreted as indirect correlates and not definitive markers. DTI captures white matter microstructural alterations along propagation pathways, revealing axonal degeneration in tracts connecting affected regions^27^. Dimensional profiling positions individual patients along a brain-first-to-body-first continuum based on the relative timing of brainstem versus cortical involvement, potentially identifying patients with distinct disease trajectories and therapeutic needs. Trajectory modeling approaches, particularly SuStaIn algorithms and network-based modeling that integrate structural and functional connectivity, may identify distinct progression pathways, reveal the earliest involvement, and predict future propagation patterns based on the current distribution of pathology.

Temporal dynamics are assessed via longitudinal changes in neuromelanin signal intensity, iron accumulation rates measured by serial QSM, atrophy progression rates quantified by volumetric analyses, and white matter degeneration rates measured by DTI^20–23,27^. Trajectory modeling approaches, including mixed-effects models and latent class growth analyses, can identify distinct progression subtypes that distinguish linear versus non-linear progression patterns and may identify inflection points where progression accelerates^25,50,57^. Dimensional profiling positions individual patients along a slow-to-fast progression continuum based on their longitudinal MRI trajectories. Insights into the temporal dynamics of disease progression may facilitate early identification of rapid progressors who require aggressive symptom management and close monitoring^45^. Once established, MRI-derived insights may be combined with clinical and genetic markers to identify patients most likely to show measurable progression within trial timeframes and to facilitate adaptive trial designs that adjust treatment based on individual progression rate^7,9,10^.

Co-pathologies are probed using white matter hyperintensities (WMH) as markers of cerebrovascular disease, medial temporal lobe atrophy suggesting concomitant Alzheimer’s pathology, and patterns of cortical atrophy that distinguish pure PD from PD with significant co-pathologies^20–23^. Dimensional profiling can quantify the relative burden of vascular versus neurodegenerative pathology in patients, with clustering methods identifying subgroups with distinct co-pathology profiles, such as “pure PD” and “PD with Alzheimer’s co-pathology^23,24,26,28^. Co-pathology profiling may partially explain prognostic heterogeneity of cognitive state and identify individuals at high risk of rapid cognitive decline and dementia requiring close monitoring and early pharmaceutical interventions^60–63^. Once established, co-pathology profiling may enable personalized prognostic counseling and inform clinical trial stratification, ensuring that cognitive interventions are tested in appropriate patient subgroups^7,9,10^.

The system response and resilience mechanisms can be investigated via measures of network connectivity, cortical thickness in regions associated with cognitive reserve, and the degree of imaging-clinical dissociation^20–23^. Resting-state functional MRI reveals compensatory network reorganization, with increased connectivity in alternative pathways that compensate for primary network dysfunction. DTI captures structural connectivity changes, while cortical thickness in the frontal and parietal association cortices is linked to cognitive reserve, predicting resilience capacity^63,68^. Dimensional profiling may quantify individual patients’ positions along a low-to-high resilience continuum, with longitudinal trajectory modeling potentially revealing whether compensatory capacity is maintained or exhausted as disease progresses^25,50,57^. Additionally, it may identify individuals with different prognoses despite similar pathology burden, aiding personalized prognostic counseling and informing clinical trial design^7,9,10^. Resilience-based stratification may be particularly valuable for preventive interventions in prodromal patients, where enhancing compensatory capacity before substantial pathology accumulates could delay or prevent clinical onset^49,57^.

## Toward MRI-derived biological subtypes

The translation of such a multidimensional classification system into clinical practice requires a coordination of methodological, validation, and regulatory steps to operationalize these dimensions, validate their biological relevance, and demonstrate clinical utility^1,2^. The first step comprises defining the operation of the dimensions and their measurable features. The conceptualized axes are biologically motivated, yet their practical implementation requires defining quantitative or categorical descriptors that capture variability along each dimension. Established biological constructs should be consistently estimated across cohorts, including clear rules for dimensional scaling, thresholds for subtype assignment when using categorical classification, and strategies for handling missing or multimodal data.

A second step is large-scale multimodal data integration and model development. The multidimensional classification framework assumes that heterogeneous biological signals can be integrated into coherent patient profiles, relying on harmonized datasets comprising imaging, clinical, genetic, and molecular biomarkers across diverse populations and disease stages. Advanced statistical approaches can model latent dimensions, identify stable subtype structures, or generate continuous biological coordinates for individual patients. Importantly, model development should emphasize robustness and interpretability rather than purely predictive performance to facilitate biological interpretation and increase the likelihood of clinical adoption.

Independent validation and reproducibility testing are essential. A classification system intended for clinical use must demonstrate stability across imaging platforms, acquisition protocols, and analytical pipelines. Multicenter replication studies are needed to assess whether the derived dimensions and subtype structures can be reproduced across diverse cohorts. Additionally, test-retest reliability and longitudinal consistency are necessary to determine whether patients maintain stable positions within the multidimensional space over relevant timeframes. Furthermore, the classification system’s sensitivity to methodological variations should be assessed, and a minimal set of biomarkers needed for reliable stratification should be identified.

The framework must demonstrate biological validity and clinical relevance. Biological validity demonstrates that the proposed dimensions correspond to meaningful underlying mechanisms, ideally supported by converging evidence from neuropathology, molecular biomarkers, or experimental models. Clinical relevance, in turn, demonstrates that patient positions within the multidimensional framework predict clinically important outcomes such as disease progression, treatment response, or development of complications. Prospective cohort studies and embedded biomarker analyses in clinical trials provide particularly valuable opportunities to test whether stratification improves prognostic accuracy or enables more targeted therapeutic strategies. Importantly, biological validity stems from validation of the proposed model across converging evidence from molecular biomarkers such as alpha-synuclein amplification assay, amyloid/tau biomarkers, inflammatory and metabolic markers, genetic profiling, PET imaging, and histopathological validation, as MRI is a component of a broader biomarker framework.

The next translational milestone is prospective clinical utility testing. For a classification system to influence clinical practice, it must improve decision-making compared with existing approaches, such as more accurate prediction of disease trajectories, improved patient selection for disease-modifying therapies, or better patient stratification for specific interventions. Such framework benefits can be demonstrated in prospective studies in which stratification results are incorporated into clinical management or trial design. Adaptive trial frameworks may be particularly well suited to evaluate whether biologically informed stratification enhances treatment effects or reduces trial heterogeneity.

Equally important are standardization and infrastructure development. Clinical implementation requires harmonized data acquisition protocols, validated analysis pipelines, and accessible computational tools capable of generating patient-level stratification outputs in routine clinical environments. International collaborations and open-science initiatives will play a central role in defining common standards for biomarker acquisition and analysis. Regulatory-grade software pipelines and transparent reporting standards are necessary to ensure that classification outputs are reproducible and interpretable across clinical sites.

Finally, the pathway toward clinical adoption requires regulatory and translational governance frameworks. Regulatory authorities increasingly recognize imaging biomarkers as potential tools for patient stratification, but their acceptance depends on rigorous evidence demonstrating analytical and clinical utilities. Engagement with regulatory agencies early in the development process can help define evidentiary requirements and appropriate validation pathways. Additionally, ethical considerations, including equitable access to advanced biomarker technologies, transparency of algorithmic decision-making, and responsible handling of patient data, must be integrated into the development process.

MRI should be viewed as one layer within a broader multimodal framework. Future directions include integrating MRI with complementary approaches to address its limitations in assessing alpha-synuclein aggregation, neuroinflammation, amyloid and tau pathology, and mitochondrial dysfunction, thereby achieving more comprehensive biological characterization.

## Conclusion

We presented a conceptual, multidimensional framework for understanding PD heterogeneity and outlined a translational roadmap to establish multidimensional MRI-based stratification. Although MRI is limited in resolving the molecular complexity of PD, it provides a non-invasive, biologically integrated, and widely available option to measure tissue integrity, spatial distribution, temporal evolution, and network-level organization. Therefore, MRI may play a substantial role in future multimodal frameworks integrated with clinical, fluid, genetic, and molecular biomarkers. Once the model is validated, the proposed framework may support biological characterization of PD heterogeneity and contribute to prognostic predictions, therapeutic decision-making, risk stratification, trial patient selection, and hypothesis generation for precision-medicine approaches. By moving beyond traditional clinical diagnosis and disease-stage classifications, such as NDS-ISS or SynNeurGe, towards a mechanistically grounded characterization of heterogeneity, a conceptual framework can be provided to understand why patients with similar diagnostic classifications may experience different clinical trajectories. Nevertheless, perspective testing and extensive validation are needed prior to clinical implementation. Significant challenges remain, including the need for standardized protocols, validation through clinicopathological correlation, and prospective demonstration of clinical utility. As MRI technology continues to advance and our understanding of PD biology deepens, MRI-based patient stratification will play an increasingly central role in transforming PD care from a one-size-fits-all approach to truly personalized medicine.

## Data Availability

No datasets were generated or analysed during the current study.
